# Development and Validation of the Assessment of Low Luminance Vision-Related Activities

**DOI:** 10.1007/s44402-026-00018-2

**Published:** 2026-02-25

**Authors:** Dinesh Venugopal, Joanne Wood, Alex Black, Chris Bradley, Sharon Bentley

**Affiliations:** 1 Centre for Vision and Eye Research, Optometry and Vision Science, School of Clinical Sciences, Queensland University of Technology, Brisbane, QLD Australia; 2 Department of Ophthalmology, Johns Hopkins University School of Medicine, Baltimore, MD USA; 3 Herbert Wertheim School of Optometry and Vision Science, University of California, Berkeley, CA USA

**Keywords:** Low luminance, Performance-based measure, Rasch analysis, Reliability, Validity

## Abstract

**Purpose:**

To develop a new comprehensive vision-related low luminance performance-based measure, the ‘Assessment of Low Luminance Vision-Related Activities’ (ALLVA), and evaluate its construct validity and test-retest reliability using Rasch analysis.

**Methods:**

A cross-sectional observational study of 75 adults with vision impairment from various ocular conditions (mean age 70 years [SD: 15 years]; mean binocular visual acuity 0.63 logMAR [SD: 0.45 logMAR]) was conducted. Seventeen tasks were developed as items and administered to participants under low luminance, with completion time and number of errors recorded. As some items could not be completed by all participants, five categories of completion time were created for analysis (quartiles and a fifth category representing non-completion). The ‘method of successive dichotomisations’—a polytomous Rasch model that always estimates ordered response category thresholds, enabling its application to binned continuous data—was applied to create a single combined measure of performance. Errors were not analysed as they occurred infrequently and generally increased completion time. Eleven participants with age-related macular degeneration were retested after 2–4 weeks. Clinical vision measures, including visual acuity, contrast sensitivity and visual fields, were also collected.

**Results:**

Initial analysis of the 17-task ALLVA led to removal of one item, walking a mobility course, due to infit and outfit mean square statistics being outside the acceptable range. For the remaining 16 tasks, item difficulty was well targeted to person ability, with only a minor floor effect. Item and person reliability values were 0.98 and 0.93, respectively. Clinical vision measures were significantly correlated with person measures. Bland–Altman analysis indicated a mean difference between test and retest person measures of −0.08 logits (95% limits of agreement 2.16 to −2.32 logits).

**Conclusion:**

The ALLVA is the first comprehensive vision-related low luminance performance-based measure. It demonstrated strong Rasch psychometric properties, validity and good test-retest reliability.

Key Points

- The Assessment of Low Luminance Vision-Related Activities (ALLVA) is the first comprehensive vision-related low luminance performance-based measure developed for a low vision population that has evidence of strong Rasch construct validity.
- Better visual acuity, contrast sensitivity and visual fields were all significantly correlated with better ALLVA person measures.
- The ALLVA may be useful in research and clinical settings to quantify performance of low luminance vision-related activities objectively, and to evaluate the outcomes of eye treatments and low vision rehabilitation.

## Introduction

Ocular diseases, such as age-related macular degeneration (AMD), glaucoma and inherited retinal disorders, have a significant effect on visual performance under low luminance conditions [1–3]. Individuals with these conditions report difficulties with night driving, walking at night, seeing steps, reading under dim illumination and adapting to changes in illumination [4, 5]. Measuring these effects has become increasingly important to better understand and quantify the difficulties experienced by patients under a range of light levels, including low luminance, and to evaluate new treatments, where improvements may be relatively small but functionally important.

Both patient-reported outcome measures (PROMs) and performance-based measures have been developed to assess the effects of vision impairment on activities of daily living [6, 7]. PROMs provide the patient perspective, are relatively low-cost and administratively efficient. However, PROMs are prone to inaccuracies and biases [8]. Performance-based measures provide a direct and more objective assessment of ability, with previous studies demonstrating the negative effects of vision impairment on the ability to perform activities of daily living under photopic conditions [7, 9].

Importantly, older adults with and without vision impairment report greater difficulties with low luminance activities and spend approximately one-third of their waking hours in these lighting conditions [10]. To date, only four low luminance PROMs have been developed and validated [11–16], and studies on low luminance performance have been limited to single activities, such as reading, face recognition, mobility and object identification [17–20]. A low luminance performance-based measure comprising a range of relevant activities that can be combined into a single measure of overall performance is needed and could be used in conjunction with PROMs and clinical vision measures.

Performance-based measures have predominantly used continuous measurements of physical attributes, typically time to complete a task and count of errors [9, 21–23]. Measurements of physical attributes have the advantage of objectivity. However, the challenge is combining performance on several items into a single overall measure of performance when not all participants can complete all items, and thus, time taken or errors cannot be measured. This can be overcome by using continuous data binned into ordinal categories and applying the method of successive dichotomisations (MSD) [24]. MSD is a polytomous Rasch model that always estimates ordered response category thresholds, enabling its application to binned continuous data. Ordinal categories for all person-item combinations are analysed to estimate the magnitude of the latent trait (ability) for each person (called the person measure) and the sensitivity to that trait of each item (called the item measure) [24].

The aim of this study was to develop a new comprehensive vision-related low luminance performance-based measure, the ‘Assessment of Low Luminance Vision-Related Activities’ (ALLVA) and to evaluate its construct validity and test-retest reliability using Rasch analysis.

## Methods

### Development of the Assessment of Low Luminance Vision-Related Activities

Items for the ALLVA were identified through a review of the literature, which included a group concept mapping study based on perspectives from individuals with vision impairment and low vision professionals [4]. In selecting tasks, consideration was given to whether the task was of high importance to individuals, could be easily standardised and representative of real-world tasks, whether it was practical to administer, safe for participants, easy to replicate and whether collectively the tasks provided a range of difficulty levels.

Seventeen tasks were selected and created as items: facial expression recognition; facial identity discrimination; sign recognition; inserting key into a lock; identification of money; matching socks; using a microwave; using a stove/oven; reading a medicine label; reading a book; searching a kitchen cupboard/counter; reading a food expiry date; reading mobile phone text; walking a mobility course; road hazard awareness (pedestrian perspective); road hazard awareness (driver perspective) and adaptation to low lighting (mobility course). Standardised instructions for each item were developed. Details for each item are provided in Online Resource Table S1, Figs. S1 and S2. Further information can be obtained from the corresponding author.

Both completion time and number of errors were recorded for each item. As expected, some participants could not complete all tasks. Therefore, for each task, a performance score was created, where time to complete a task was categorised into quartiles with a fifth category representing non-completion (refer to Online Resource Table S3 for time thresholds and the number of participants who did not complete each item). Errors were not analysed as a measure of performance, because, consistent with Gobeille et al. [22], errors occurred infrequently and there was a ceiling effect. Furthermore, a plot of the number of errors against time taken for each individual item and linear regression analysis indicated a positive slope, meaning that errors were generally associated with a longer completion time for all tasks.

The ALLVA was pilot tested on seven participants with normal vision under low luminance to evaluate feasibility, safety, clarity and burden (see Online Resource Table S2). Five completed the ALLVA a second time with simulated vision impairment (visual field constriction ~10° in diameter). Only minor modifications to clarify instructions for some of the items were needed. The ALLVA took a mean of 26.0 min (SD: 0.7 min) for participants with normal vision to complete and 31.7 min (SD: 2.6 min) for participants with simulated vision impairment.

### Participants

Participants were recruited from the Queensland University of Technology optometry clinic, Glaucoma Australia and Retina Australia.

Eligible participants were aged 18 years or older, with any ocular disease, habitual visual acuity (VA) of 6/12 or worse (≥0.30 logMAR) in each eye and/or the presence of any visual field defect in both eyes (one or more points *P* < 5.0% on Humphrey Field Analyzer [HFA] 24-2 total deviation plot). Exclusion criteria were difficulty hearing and self-reported or clinical history of cognitive or mobility impairment.

A sub-sample of participants with AMD performed the ALLVA on a second occasion to determine test-retest reliability. Inclusion criteria were the same as described above, with the additional criterion of having stable AMD, indicated by no reported changes in vision and no more than ±0.14 logMAR difference in VA on the two test occasions [25]. Participants undergoing treatment for AMD were excluded.

### Data Collection and Procedure

Demographic data included age and gender. Ocular history, general health history and medications were recorded. Photopic clinical vision measures were also obtained. Binocular distance VA was measured with habitual spectacle correction using an Early Treatment Diabetic Retinopathy Study chart [26]. A termination rule of four or more errors on a line and per letter scoring was used [27]. Binocular peak contrast sensitivity (CS) was measured with near habitual spectacle correction using the Melbourne Edge Test [28], with a termination rule of two consecutive errors. For participants with ocular disease affecting peripheral vision, visual fields were assessed monocularly using HFA 24-2 Swedish Interactive Threshold Algorithm (SITA) standard mean deviation (MD).

The ALLVA was administered by one researcher (DV) under mesopic conditions of ~0.2 cd/m^2^ following 10 min of adaptation to the light level. Participant performance was video-recorded.

To evaluate test-retest reliability of the ALLVA, the sub-sample of participants with AMD was retested 2–4 weeks after the first test by the same researcher (DV) [8]. Photopic distance VA was also measured at the second session to confirm vision was stable.

### Data Analysis

Rasch models analyse ordinal categorical data to estimate measures of the latent trait (in this case, ability to perform vision-related low luminance activities) on an equal interval scale in units of logit (log-odds). They estimate the relative difficulty of items (item measures), relative abilities of participants (person measures), and the locations of response category thresholds—boundaries between neighbouring response categories that define the ordinal categorical variable (e.g., Likert-type scales). MSD is currently the only polytomous Rasch model that always estimates ordered response category thresholds, making it suitable to apply to the continuous data binned into the five-level ordinal scale described in the section on the development of the ALLVA above. The R package ‘msd’ (R Foundation for Statistical Computing, r-project.org) was used to estimate item measures, person measures and thresholds. ALLVA measures were estimated on a scale such that negative person measures indicated more visual ability (i.e., the participant completed the task quickly), and negative item measures indicated more difficult tasks.

Applying MSD to performance measures also meant that traditional Rasch analysis statistics could be used to assess the psychometric properties of ALLVA. First, infit and outfit mean square (MNSQ) statistics were used to assess the unidimensionality of ALLVA (i.e., that items measure a single trait). The infit statistic is less sensitive to outliers and considered more informative than the outfit statistic [29]. The infit MNSQs compare the observed variance in the responses to the expected, with values of 1.0 indicating unidimensionality. MNSQ values between 0.5 and 1.7 logits are considered acceptable for clinical observations [30, 31]. Items and persons with MNSQs < 0.5 (both infit and outfit) suggest redundancy. Items with MNSQs > 1.7 (both infit and outfit) are diagnosed as misfitting and removal should be considered. Persons with infit MNSQs > 1.7 suggest unexpected responses and are diagnosed as misfitting. Additionally, the distribution of person infit MNSQ was compared to a weighted sum of chi-square divided by degrees of freedom to evaluate overall fit. Under ideal measurement conditions, the underlying measurement error should be normally distributed, and the mean of the person infit MNSQ values should be ~1.0. Unidimensionality was also evaluated by applying principal components analysis to the binned time data.

Targeting was assessed by comparing the distribution of item measures and person measures (there should be no ceiling or floor effect). Precision was evaluated using item reliability and person reliability statistics (values should be >0.9 and >0.8, respectively) [29]. Additionally, standard errors were estimated for item and person measures, with smaller values indicating greater precision [32]. Standard errors were plotted against item and person measures, with the expected distribution being a characteristic U-shaped pattern with good targeting, reflecting increased uncertainty (larger standard error) at the extreme ends of the measurement scale.

Item difficulty should be similar for persons at the same level of ability, regardless of age or gender group. Differential item functioning (DIF) measures for each item were obtained using the R ‘lordif’ package for age (<73 years vs. ≥73 years, based on the median age of the sample) and gender. [33] Uniform DIF (appropriate given the use of MSD) was assessed considering both statistical significance and effect size, using the *χ*^2^ and pseudo-R-square statistics. An item was considered to exhibit meaningful DIF if the *p*-value was <0.001 and the absolute value of the log odds ratio was >0.64.

To evaluate convergent validity, correlations between ALLVA person measures (in logits) and photopic VA, CS and better eye HFA MD were calculated using Pearson’s correlation coefficients.

Bland–Altman analysis was used to evaluate the test-retest reliability of ALLVA person measures for the 11 AMD participants who completed testing at two different study visits [34, 35]. MSD was separately applied to 86 data sets (75 from the participants with vision impairment who completed the ALLVA once and 11 from the participants with AMD who completed a retest). Person measures for the 11 participants with AMD who completed the ALLVA twice were extracted to examine test-retest reliability. The mean difference between test and retest person measures and the coefficient of repeatability (1.96 × SD of mean differences between two measurements) were calculated [35]. A plot of the difference between the test-retest ALLVA person measures against the mean of the test-retest ALLVA person measures, with test-retest 95% limits of agreement (LoA; mean test–retest difference ±1.96 SD) and confidence limits (using the exact method) [36, 37], was evaluated. For comparison with other studies, an intraclass correlation coefficient (ICC; two-way mixed effect, absolute agreement) and 95% confidence interval (CI) was calculated [8, 38–40].

## Results

### Participant Characteristics

Seventy-five individuals with vision impairment participated (characteristics provided in Table 1).

**Table 1:** Participant characteristics (*n* = 75).

Characteristic	Mean (SD) or *n* (%)
Age (years)	70.0 (15.2)
Gender	
Women	38 (51%)
Men	37 (49%)
Visual acuity (logMAR)	0.63 (0.45)
Contrast sensitivity (dB)	14.4 (5.2)
Better eye HFA 24-2 MD (dB)^a^	−9.56 (9.16)
Cause of vision impairment	
Age-related macular degeneration	24 (32%)
Glaucoma	15 (20%)
Macular dystrophy	8 (10%)
Ocular albinism	6 (8%)
Cone dystrophy	3 (4%)
Optic neuropathy	3 (4%)
Retinitis pigmentosa	2 (3%)
Retinal detachment	2 (3%)
Combined causes of vision impairment (e.g., maculopathy and retinitis pigmentosa)	4 (5%)
Other ocular diseases	8 (10%)

*HFA* Humphrey Field Analyzer, *MD* mean deviation.

^a^*n* = 22 (participants with peripheral vision impairment only; MD range = −0.33 to −27.48 dB).

### Rasch Construct Validity

For one participant with a diagnosis of glaucoma, it was not possible to estimate the MSD person measure, MNSQ and standard error (visual acuity −0.10 logMAR, better eye HFA 24-2 MD −0.35 dB) because completion time for all items was in the first quartile (technically, the person measure for this participant is +∞). Therefore, all subsequent analyses were limited to the remaining 74 participants with vision impairment, which resulted in a small change in mean HFA 24-2 MD for the sample (mean MD: −10.00 dB, SD: 9.15 dB) and no change in the range. The item infit MNSQs ranged from 0.59 to 2.89 logits, with two items (walking a mobility course and reading a book) outside the acceptable range of 0.5 to 1.7. For walking a mobility course (item measure 0.84 logits), both the infit and outfit MNSQ statistics (1.75 and 1.83 logits, respectively) indicated misfitting. Although mobility is an important activity of daily living, constructing an indoor mobility course that incorporates a range of real-world challenges is complex, in part due to safety constraints and requires a reasonable amount of space. As ALLVA includes another shorter, simpler mobility task (adaptation to low lighting [mobility course]) of similar difficulty (item measure 0.84 logits) with an infit MNSQ statistic within the desired range, the decision was made to remove the more complex mobility course item. For reading a book, only the infit MNSQ (2.89 logits) was outside the acceptable range. At this stage, given its importance to patients, the decision was made to retain the item. The person infit MNSQ for the initial analysis ranged between 0.22 and 2.66 logits, with 78% of the persons inside the acceptable range of 0.5 to 1.7. Items were well targeted to persons, with only a minor floor effect. The item and person reliabilities of the ALLVA were 0.98 and 0.93, respectively, indicating good precision. The mean standard error of the item measures was 0.21 logits (SD: 0.04, range: 0.18–0.31 logits), and the mean standard error of the person measures was 0.49 logits (SD: 0.10, range: 0.33–0.78 logits).

MSD analysis was repeated for the 16-task ALLVA. Item fit statistics are provided in Table 2. The item infit MNSQ ranged between 0.60 and 3.11 logits, with all items within the acceptable range of 0.5 to 1.7, except reading a book, which was the most difficult item. Again, the decision was made to retain the item, given its relevance and importance to patients and because it extends the range of the ALLVA measurement. Furthermore, removal of the reading a book item resulted in slightly poorer targeting and reliability of the ALLVA. All 16 items had acceptable outfit MNSQs, except the item, identification of money, which was marginally outside the acceptable range.

**Table 2:** Item fit statistics.

Items	Infit MNSQ (logits)	Outfit MNSQ (logits)
Facial expression recognition	0.68	0.64
Facial identity discrimination	1.12	1.05
Sign recognition	0.60	0.58
Inserting key into a lock	0.81	0.78
Identification of money	1.55	1.92^a^
Matching socks	1.11	1.24
Using a microwave	0.90	0.82
Using a stove/oven	1.15	0.88
Reading a medicine label	1.46	1.04
Reading a book	3.11^a^	1.63
Searching a kitchen cupboard/counter	0.65	0.70
Reading a food expiry date	0.70	0.65
Reading mobile phone text	1.10	0.94
Road hazard awareness: Pedestrian perspective	1.30	1.43
Road hazard awareness: Driver perspective	1.00	1.18
Adaptation to low lighting (mobility course)	1.04	1.05

*MNSQ* mean-square.

^a^Misfitting item.

**Fig. 1:**
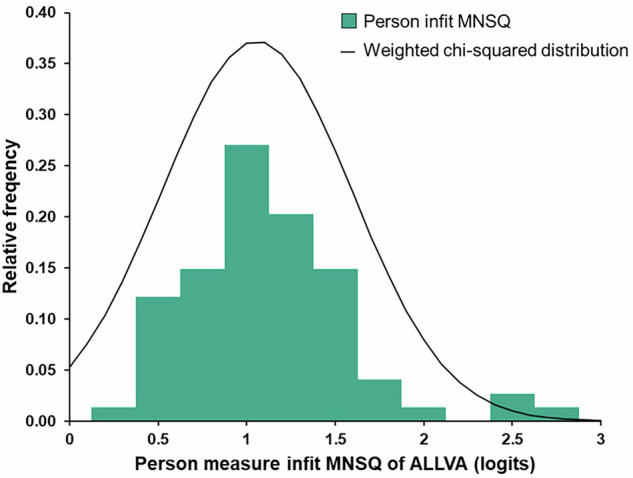
Person infit mean square (MNSQ) and expected weighted sum of chi-squared distribution by its degrees of freedom (black curve) of Assessment of Low Luminance Vision-Related Activities (ALLVA). Person infit MNSQ (teal bars) are plotted relative to a weighted sum of chi-squared distribution (black line). Most person infit MNSQ fall within the weighted chi-squared distribution and around the expected value of 1.0, indicating an approximately normal distribution.

The person infit MNSQ ranged between 0.28 and 2.82 logits, with 73% of the persons within the acceptable range of 0.5 to 1.7. Figure 1 shows a histogram of person infit MNSQ and a weighted sum of chi-square/degrees of freedom distributions, indicating an approximately normal distribution. Most person infit MNSQs fall within the weighted chi-squared distribution and around the expected value of 1.0, indicating an approximately normal distribution.

The proportion of item and person infit MNSQs within the acceptable range suggests the ALLVA is unidimensional.

Dimensionality of the 16-task ALLVA was also explored using principal component analysis. The first component explained 60.1% of the variance, the next residual component explained 8.2% of the variance, and all remaining components explained less than 5.4% of the variance, indicating unidimensionality of the instrument.

**Fig. 2:**
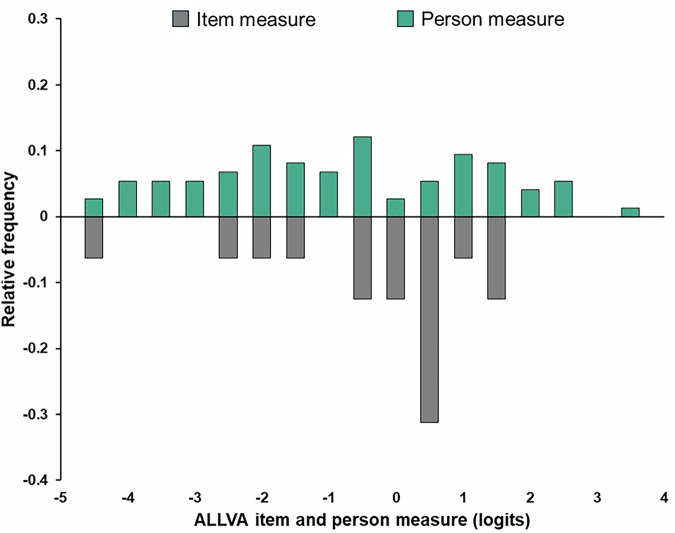
Wright map for Assessment of Low Luminance Vision-Related Activities (ALLVA). Item measures (grey bars) and person measures (teal bars) show good overlap for ALLVA. Negative item measures indicate more difficult items, and negative person measures represent participants with more visual ability (i.e., completed the tasks quickly).

Item measures are provided in Table 3. A Wright map (Fig. 2) showed that ALLVA items were well targeted to persons, with the item measurement range spanning 5.99 logits, from −4.06 logits for the most difficult item (reading a book) to 1.93 logits for the least difficult item (road hazard awareness [pedestrian perspective]). However, three items overlapped at 0.96 logits (inserting key into a lock, matching socks and adaptation to low lighting mobility course), and a minor floor effect was evident (i.e., no easy items beyond 2.00 logits; Fig. 2).

**Table 3:** Item measures.

Items	Item measures (logits)
Reading a book	−4.06
Reading a medicine label	−2.31
Using a stove/oven	−1.85
Reading mobile phone text	−1.04
Reading a food expiry date	−0.47
Using a microwave	−0.19
Facial identity discrimination	0.02
Facial expression recognition	0.41
Sign recognition	0.58
Inserting key into a lock	0.96
Matching socks	0.96
Adaptation to low lighting (mobility course)	0.96
Searching a kitchen cupboard/counter	1.00
Road hazard awareness: Driver perspective	1.36
Identification of money	1.73
Road hazard awareness: Pedestrian perspective	1.93

**Fig. 3:**
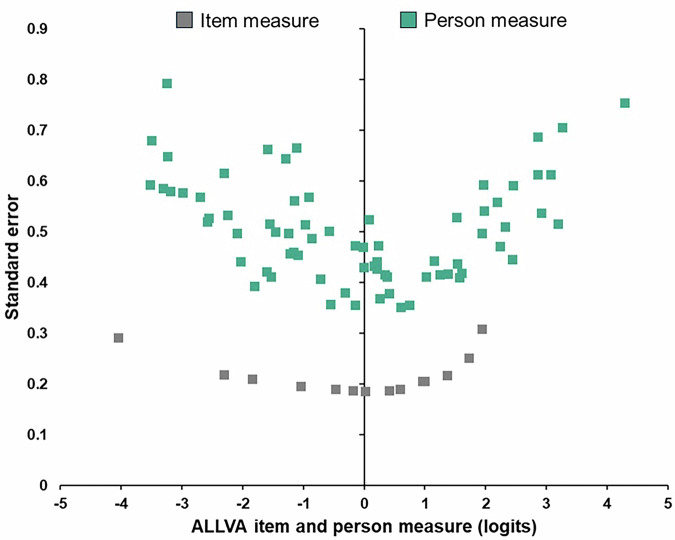
Item and person measure standard errors of 16-task ALLVA plotted against their relative item (grey) and person measures (teal). Negative item measures indicate more difficult items, while negative person measures represent participants with more visual ability (i.e., completed the tasks quickly). Note, there are some overlapping data points. ALLVA Assessment of Low Luminance Vision-Related Activities.

Item and person reliability values for the 16-item ALLVA were 0.98 and 0.93, respectively. The mean standard error of the item measures was 0.22 logits (SD: 0.04, range: 0.19–0.31 logits), while the mean standard error of the person measures was 0.51 logits (SD: 0.10, range: 0.35–0.79 logits). Item and person measure standard errors were plotted against their respective item and person measures (Fig. 3), revealing U-shaped distributions, as expected for well-targeted instruments, with greater standard errors for extreme item and person measures than those in between.

None of the items in ALLVA met the criteria for statistically significant DIF (*p* < 0.001) or exceeded the log odds ratio threshold of 0.64 for both age (<73 years [*n* = 36] vs. ≥73 years [*n* = 39]) and gender (women [*n* = 37] vs. men [*n* = 38]), indicating that there was no evidence of item biases.

### Convergent Validity

Correlations between various vision measures and ALLVA person measures are provided in Table 4. Better VA, CS and visual fields were all significantly correlated with better ALLVA person measures, with the correlation for CS being strongest (*r* = −0.82).

**Table 4:** Correlations between vision measures and ALLVA person measures (*n* = 74)^a^

	Visual acuity (logMAR)		Contrast sensitivity (dB)		HFA MD of better eye (dB)^b^	
	*r*	*p* value	*r*	*p* value	*r*	*p* value
ALLVA person measure (logits)	0.74	<0.001	−0.82	<0.001	−0.53	0.01

*ALLVA* Assessment of Low Luminance Vision-Related Activities, *dB* decibels, *HFA* Humphrey Field Analyzer, *MD* mean deviation.

^a^Pearson correlation coefficient.

^b^*n* = 21 (assessed only in participants with peripheral vision impairment).

### Test-Retest Reliability

Eleven adults with AMD participated in the evaluation of test-retest reliability, with a mean age of 79.1 years (SD: 6.3 years) and 73% male. The median time between the test and retest sessions was 3.4 weeks (range: 2.0 to 4.0 weeks).

**Fig. 4:**
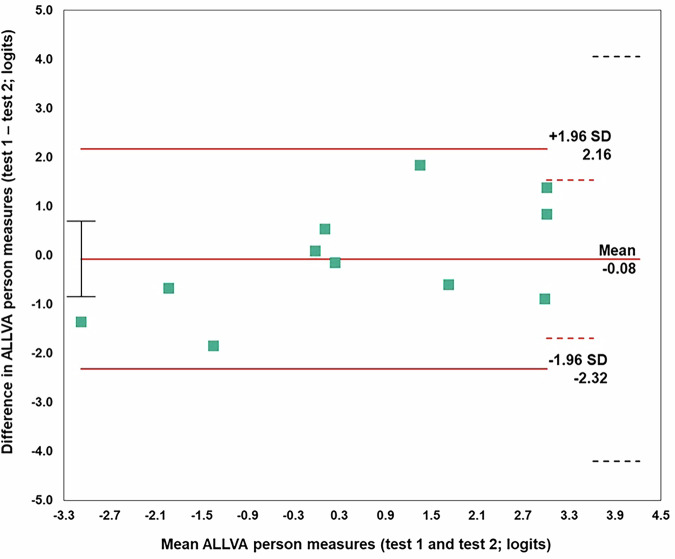
Bland–Altman plot for total Assessment of Low Luminance Vision-Related Activities (ALLVA) person measures (logits), (*n* = 11). Red lines represent mean of differences and limits of agreements (LoAs), black error bar represents 95% confidence interval for mean of differences, black dotted lines represent the outer (97.5%) confidence limits of LoAs and red dotted lines represent the inner (2.5%) confidence limits of LoAs.

The mean test-retest difference in the ALLVA person measure was −0.08 logits (95% CI, −0.85 to 0.69; SD = 1.14 logits), which was not significantly different from zero (*p* = 0.83), indicating the absence of any systematic bias (Fig. 4). The upper and lower LoAs were 2.16 and −2.32 logits, respectively, and the coefficient of repeatability was 3.17 logits. The ICC was 0.92 (95% CI, 0.69–0.98).

## Discussion

A new comprehensive vision-related low luminance performance-based measure, the ALLVA, was developed and validated using MSD Rasch analysis. Of the initial 17 items, one misfitting item was removed (walking a mobility course). The 16-task ALLVA exhibited unidimensionality and good precision. One item (reading a book) was misfitting based on infit MNSQ; however, it was retained due to its relevance and importance to patients. Item difficulty was very well targeted to person's ability with only a minor floor effect. Clinical measures of vision were moderately to strongly correlated with ALLVA person measures. The mean test-retest difference was close to zero. Overall, the ALLVA demonstrated good validity and test-retest reliability.

Following initial analysis, walking a mobility course was removed due to misfitting, with fit statistics suggesting inconsistent performance of the item across participants. This could have been due to less than one quarter of the sample having ocular conditions that reduce mobility performance, such as retinitis pigmentosa and glaucoma [41,42], resulting in most participants finding the task relatively easy. For safety reasons, the course was intentionally constructed with low-risk obstacles. Regardless, the item was partially redundant with a shorter, simpler mobility task, adaptation to low lighting (mobility). Subsequent analysis demonstrated that removal of the walking mobility course item did not compromise the overall measurement quality of the ALLVA.

For the 16-item ALLVA, the distribution of person measure infit MNSQs and the principal components analysis indicated that the instrument was unidimensional (measuring a single underlying trait). The number of misfitting items in the 16-item ALLVA was fewer than in previous studies of similar instruments [9,43,44]. However, some other studies intentionally began with a large number of items, using Rasch analysis to eliminate unsuitable items; whereas the current study used a more targeted approach to identify items from both low vision patients and professionals [4]. Reading a book did not fit the Rasch model based on a high infit MNSQ. High item infit MNSQs indicate unexpected inlier performances; performances that fall within the expected range of a participant’s ability or an item’s difficulty but deviate from the pattern expected by the Rasch model. Reading a book was the most difficult item, and over 70% of participants were unable or did not make an attempt to complete it. The item comprised a long passage of text that took a long time to complete, even for people with normal vision, and performance could have been influenced by fatigue. Given the relevance and importance of reading books [4], the item was retained. However, future studies should consider reducing the length of the reading of a book item and re-evaluating its fit.

Targeting was very good for the sample, with only a minor floor effect due to there being no ‘easy’ items beyond 2.0 logits. It should be noted that targeting could be affected if time threshold distributions differ across patient cohorts, in which case item measures should be re-estimated using the methods outlined in this study. In this sample, there was a relatively large gap between the two most difficult items: reading a book and reading a medicine label. Noting that targeting can be sample dependent, testing and incorporating a few easier items, as well as a few harder items to fill the gap at the other end of the scale, could be an option in the future. However, it is challenging to conceive of items that would be between the two most difficult items and regardless, it is likely that the gap in difficulty would decrease if reading a book were to be reduced in length, as proposed. Three items (inserting the key into a lock, matching socks and adaptation to low lighting) had the same level of difficulty. Future studies should evaluate whether one or two of these items could be removed to reduce the administrative and participant burden without compromising the strong psychometric properties of the ALLVA.

The moderate to strong correlations between photopic clinical vision measures and the ALLVA score provide evidence of convergent validity. As found in previous studies of performance of photopic vision-related activities [45,46], better CS was more strongly correlated with better performance of low luminance vision-related activities than VA, although the difference was small. Understanding whether low luminance clinical vision measures are even better correlated with the performance of low luminance vision-related activities than photopic clinical vision measures would be of value.

The test-retest reliability of the ALLVA was reasonably good for participants with AMD, as the mean difference in scores between the two occasions was not significantly different from zero and did not vary systematically across the range of performance measured. However, the 95% LoAs were relatively wide (2.16 to −2.32 logits), in part due to the relatively small sample size. For context, performance on the 16-task ALLVA ranged from −2.39 to 3.43 logits for the test session and ranged from −3.75 to 3.43 logits for the retest session. Clinical interpretation is challenging in the case of a new measurement instrument [34], and whether the LoAs found for the ALLVA are clinically acceptable will require further study. The ICC for the ALLVA (0.92) was greater than the minimum ICC value considered acceptable for test-retest reliability (0.80) [6,47]. For comparison, a similarly high ICC has been reported for three relevant vision-related instruments [9,11,21,48,49], ranging from 0.82 for the emotional subscale of the Vision Impairment in Low Luminance questionnaire to 0.98 for the ‘Very Low Vision Instrumental Activities of Daily Living’ performance-based measure.

Outcome measures that meaningfully measure visual ability in real life, and that are informative for researchers, clinicians and patients are needed. Others have shown that self-reports should not be used as a proxy for actual task performance in people with low vision, with weak to moderate correlations between the two, and have recommended using an actual performance measure to complement self-reports in clinical trials [22,50]. Likewise, this study and others [50] have demonstrated that clinical vision measures (e.g., VA, CS and visual field) do not provide a complete picture of visual ability and should not be used as a proxy for actual task performance. However, measuring actual performance in the real world has its challenges; in particular, variable lighting and contrast. The ALLVA developed in this study fills a gap in the field by providing a single outcome measure for low luminance visual performance in clinical trials, where the person measures outcome reflects performance on a comprehensive set of standardised tasks close to real-life scenarios. Person measures from the ALLVA could be used to monitor changes in performance with disease progression or pre- and post-intervention following treatment or low vision rehabilitation. A person would be able to move from less difficult items to more difficult ones, corresponding to a shift in their person measure, as an indicator of an effective intervention. A shift exceeding the 95% confidence interval (1.96 times the standard error of the person measure estimate) would represent a statistically significant change.

There are some limitations of this study that should be considered. It should be noted that Rasch analysis weights each individual item equally and should not be applied to combining individual items where it might be desirable to weight each differently. Although the items in the ALLVA were based on perspectives from patients and professionals, and a broad review of the literature, the content and calibrations are specific to local culture and language. Additionally, the sample size in this study was at the lower end of the range recommended by Linacre [29] of 64 to 144. Most of the participants had AMD or glaucoma. It is important that a larger sample of individuals with retinitis pigmentosa and other genetic eye diseases are included in future studies, given that many emerging treatments are targeted to these conditions. Likewise, test-retest reliability should be investigated in a larger group that includes participants with eye conditions other than AMD. Also, other forms of validity and responsiveness to change after patients have undergone treatments should be investigated.

In conclusion, the ALLVA is the first comprehensive vision-related low luminance performance-based measure to be developed and validated. Importantly, the perspectives of both patients and low vision professionals, as well as a literature review, informed the items comprising the ALLVA. This study has demonstrated that the 16-task ALLVA has strong Rasch psychometric properties, convergent validity and good test-retest reliability when applied to a sample of individuals with vision impairment.

## Supplementary Information

Supplementary materials

## Data Availability

Data are available upon reasonable request to the corresponding author.
