# Supplementary material for: Development and Validation of the Assessment of Low Luminance Vision-Related Activities
Source: Ophthalmic Physiol Opt. 2026 Feb 25;46(1):81–9. doi: 10.1007/s44402-026-00018-2 (PMC13369677; doi:10.1007/s44402-026-00018-2)
Supplement: Supplementary file 1 — Supplementary materials [file 44402_2026_18_MOESM1_ESM.docx]

**Supplement table S1.** Description of the ‘Assessment of Low Luminance Vision-Related Activities’.

| **Items** | **Item description** | **Administration** | **Working distance** | **Rationale** |
| --- | --- | --- | --- | --- |
| 1. Facial expression recognition | Name the expression (happy or angry) for single faces (life-size at 1 m); two images of the same female face from the Karolinska Directed Emotional Faces database presented sequentially, one with a happy expression and one with an angry expression (identification number AF23HAS and AF23ANS, respectively) [[1](#_ENREF_1), [2](#_ENREF_2)]. | Desk-based | 1 m | Literature [[3-5](#_ENREF_3)] and GCM study [[6](#_ENREF_6)] |
| 1. Facial identity discrimination | Select the odd face out of three faces (life-size at 3 m); two sets of three faces side-by-side from the Karolinska Directed Emotional Faces database presented sequentially, (set one identification numbers AF06NES, AF11NES [odd face], AF06NES and set two identification numbers AM31NES, AM31NES, AM06NES [odd face]) [[1](#_ENREF_1), [2](#_ENREF_2)]. | Desk-based | 1 m |  |
| 1. Sign recognition | Identify either a male or female toilet sign placed next to each other. | Desk-based | 1 m | GCM study [[6](#_ENREF_6)] |
| 1. Inserting key into a lock | Insert key into a mounted lock unit. | Desk-based | 25-40 cm | GCM study [[6](#_ENREF_6)] |
| 1. Identification of money | Select set amount of money from a pile of coins (comprising two $2, two $1, two 20¢, one 10¢, and one 5¢). | Desk-based | 25-40 cm | Literature [[7-11](#_ENREF_7)] and GCM study [[6](#_ENREF_6)] |
| 1. Matching socks | Match pairs of same-coloured socks (white, grey, dark blue). | Desk-based | 25-40 cm | Literature [[12](#_ENREF_12)] and GCM study [[6](#_ENREF_6)] |
| 1. Using a microwave | Set a specific time on a simulated microwave panel. | Desk-based | 25-40 cm | GCM study [[6](#_ENREF_6)] |
| 1. Using a stove/oven | State temperature indicated on a stove dial. | Desk-based | 25-40 cm | GCM study [[6](#_ENREF_6)] |
| 1. Reading a medicine label | Read out instructions and dosage on a medicine label. | Desk-based | 25-40 cm | Literature [[7-10](#_ENREF_7), [13](#_ENREF_13)] and GCM study [[6](#_ENREF_6)] |
| 1. Reading a book | Read book size print (IReST chart) [[14](#_ENREF_14)]. | Desk-based | 25-40 cm | Literature [[3](#_ENREF_3), [5](#_ENREF_5), [12](#_ENREF_12), [15](#_ENREF_15)] and GCM study [[6](#_ENREF_6)] |
| 1. Searching a kitchen cupboard/counter | Find and set aside a particular food item from among other food items. | Desk-based | 25-40 cm | Literature [[3](#_ENREF_3), [12](#_ENREF_12)] and GCM study [[6](#_ENREF_6)] |
| 1. Reading a food expiry date | Find and read out the expiry date on a milk bottle. | Desk-based | 25-40 cm | GCM study [[6](#_ENREF_6)] |
| 1. Reading mobile phone text | Read out an appointment text message on a mobile phone. | Desk-based | 25-40 cm | GCM study [[6](#_ENREF_6)] |
| 1. Walking a mobility course^†^ (removed in final version) | Walk a 28.4 m indoor course along three sides of a room with obstacles (simulating cracks in pavement, shadows/holes, curbs) and stepping tasks (see figure S1). | Indoors | - | Literature [[3](#_ENREF_3), [5](#_ENREF_5), [12](#_ENREF_12), [16](#_ENREF_16)] and GCM study [[6](#_ENREF_6)] |
| 1. Road hazard awareness: Pedestrian perspective | Identify an oncoming car in a video-based real-world scenario (scenario staged with a single unambiguous hazard recorded on a quiet two-way suburban street at night). | Computer-based | 60-65 cm | GCM study [[6](#_ENREF_6)] |
| 1. Road hazard awareness: Driver perspective | Identify a pedestrian in a video-based real-world scenario (scenario staged with a single unambiguous hazard recorded on a quiet two-way suburban street at night). | Computer-based | 60-65 cm | Literature [[5](#_ENREF_5), [12](#_ENREF_12), [15](#_ENREF_15), [17](#_ENREF_17), [18](#_ENREF_18)] and GCM study [[6](#_ENREF_6)] |
| 1. Adaptation to low lighting (mobility course)^‡^ | Walk a straight 6.6 m path with obstacles and stepping tasks (see figure S2), immediately following sudden dimming of lights. | Indoors | - | Literature [[5](#_ENREF_5), [15](#_ENREF_15), [16](#_ENREF_16), [19](#_ENREF_19), [20](#_ENREF_20)] and GCM study [[6](#_ENREF_6)] |

GCM, group concept mapping.

^†^ See Supplement figure S1.

^‡^ See Supplement figure S2.

To set-up and administer ‘Assessment of Low Luminance Vision-Related Activities’, contact the authors for further specific details.


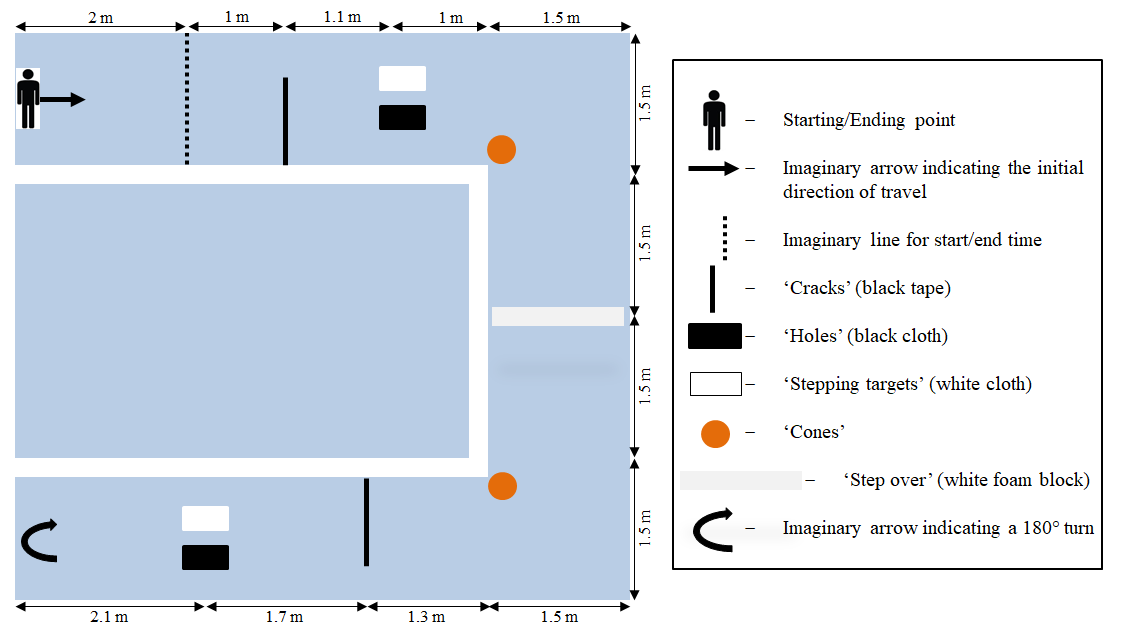


**Supplement figS1:** Layout for item 14 ‘walking a mobility course’.


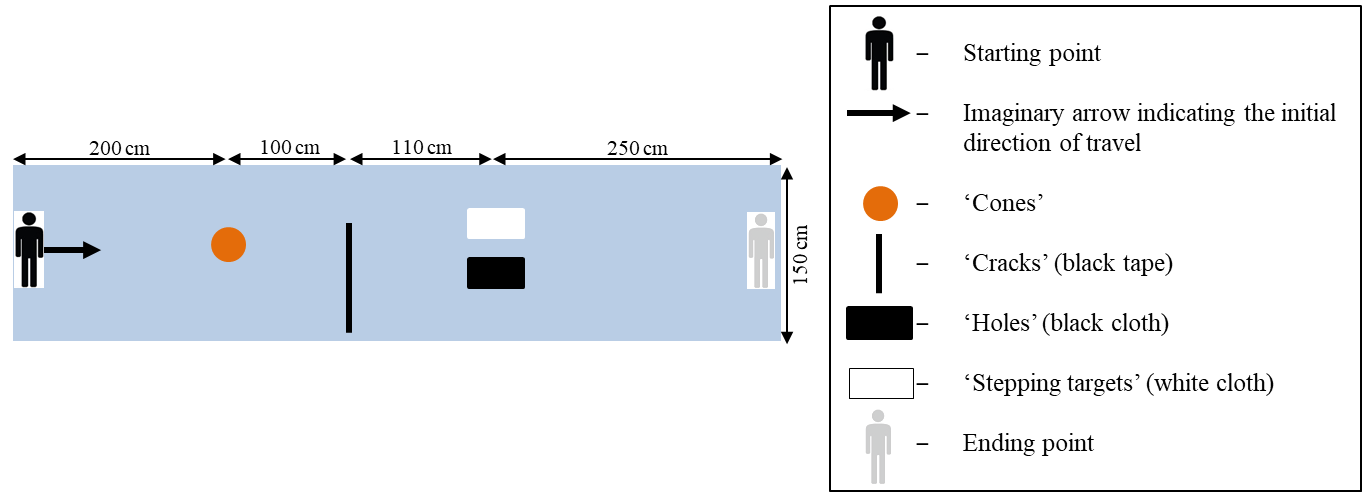


**Supplement figS2:** Layout for item 17 ‘adaptation to low lighting (mobility course)’.

**Supplement table S2.** Normative data: Mean time taken (seconds) for Assessment of Low Luminance Vision-Related Activities items (n = 7 normal vision^†^).

| Items | Mean time (SD) in seconds |
| --- | --- |
| 1. Facial expression recognition | 2.09 (0.35) |
| 1. Facial identity discrimination | 3.95 (0.68) |
| 1. Sign recognition | 1.25 (0.43) |
| 1. Inserting key into a lock | 1.72 (0.59) |
| 1. Identification of money | 5.11 (1.03) |
| 1. Matching socks | 4.85 (2.22) |
| 1. Using a microwave | 3.68 (1.39) |
| 1. Using a stove/oven | 3.29 (2.79) |
| 1. Reading a medicine label | 3.02 (0.55) |
| 1. Reading a book | 50.21 (4.16) |
| 1. Searching a kitchen cupboard/counter | 3.99 (1.25) |
| 1. Reading a food expiry date | 2.34 (0.61) |
| 1. Reading mobile phone text | 10.11 (1.25) |
| 1. Walking a mobility course (removed in final version) | 22.47 (2.17) |
| 1. Road hazard awareness: Pedestrian perspective | 1.04 (0.46) |
| 1. Road hazard awareness: Driver perspective | 1.71 (0.37) |
| 1. Adaptation to low lighting (mobility course) | 6.00 (0.81) |

SD, standard deviation.

^†^ Mean age 35.0 ± 25.1 years.

**Supplement table S3.** Time thresholds (seconds) for Assessment of Low Luminance Vision-Related Activities items (n = 75).

| Items | Q1^†^ | Q2^†^ | Q3^†^ | Q4^†^ | Number of participants who did not complete^‡^ |
| --- | --- | --- | --- | --- | --- |
| 1. Facial expression recognition | 2.95 | 4.63 | 9.23 | 28.56 | 13 |
| 1. Facial identity discrimination | 6.46 | 10.12 | 13.47 | 27.76 | 18 |
| 1. Sign recognition | 1.80 | 2.89 | 4.66 | 12.40 | 11 |
| 1. Inserting key into a lock | 2.63 | 4.19 | 7.50 | 47.44 | 0 |
| 1. Identification of money | 10.03 | 17.50 | 28.72 | 131.66 | 2 |
| 1. Matching socks | 7.13 | 9.65 | 13.57 | 36.25 | 0 |
| 1. Using a microwave | 8.08 | 13.93 | 20.26 | 37.94 | 20 |
| 1. Using a stove/oven | 4.13 | 6.16 | 12.09 | 42.50 | 38 |
| 1. Reading a medicine label | 5.13 | 7.38 | 10.28 | 30.25 | 42 |
| 1. Reading a book | 77.05 | 97.29 | 128.02 | 199.56 | 53 |
| 1. Searching a kitchen cupboard/counter | 5.91 | 9.59 | 14.53 | 63.19 | 0 |
| 1. Reading a food expiry date | 3.95 | 7.17 | 13.08 | 47.35 | 23 |
| 1. Reading mobile phone text | 14.69 | 17.47 | 25.12 | 65.72 | 30 |
| 1. Walking a mobility course (removed in final version) | 24.67 | 31.25 | 37.83 | 52.41 | 0 |
| 1. Road hazard awareness: Pedestrian perspective | 1.81 | 3.83 | 8.38 | 15.36 | 1 |
| 1. Road hazard awareness: Driver perspective | 2.97 | 4.47 | 5.82 | 9.31 | 4 |
| 1. Adaptation to low lighting (mobility course) | 7.35 | 9.47 | 11.94 | 57.19 | 0 |

Q1, first quartile; Q2, second quartile; Q3, third quartile; Q4, fourth quartile.

^†^ Participants who completed the task within Q1 were assigned to category 1; those within Q1 and Q2 to category 2; Q2 and Q3 to category 3; and Q3 and Q4 to category 4 on the five-level ordinal scale.

^‡^ Assigned category 5 on the five-level ordinal scale.

**REFERENCES**

1. Lundqvist D, Flykt A, Öhman A. The Karolinska directed emotional faces - KDEF. Cogn Emot. 1998.

2. Venugopal D, Wood JM, Black AA, Bentley SA. Effect of low luminance on face recognition in adults with central and peripheral vision loss. Ophthalmic Physiol Opt. 2023;43(6):1344-55. <https://doi.org/10.1111/opo.13198>.

3. Bijveld MM, van Genderen MM, Hoeben FP, Katzin AA, van Nispen RM, Riemslag FC, et al. Assessment of night vision problems in patients with congenital stationary night blindness. PLoS One. 2013;8(5):e62927. <https://doi.org/10.1371/journal.pone.0062927>.

4. Bullimore MA, Bailey IL, Wacker RT. Face recognition in age-related maculopathy. Invest Ophthalmol Vis Sci. 1991;32(7):2020-9.

5. Pondorfer SG, Terheyden JH, Overhoff H, Stasch-Bouws J, Holz FG, Finger RP. Development of the vision impairment in low luminance questionnaire. Transl Vis Sci Technol. 2021;10(1):5. <https://doi.org/10.1167/tvst.10.1.5>.

6. Bentley SA, Black AA, Hindmarsh GP, Owsley C, Wood JM. Concept mapping to identify content for a performance-based measure of low luminance vision-related activities of daily living. Transl Vis Sci Technol. 2022;11(9):27. <https://doi.org/10.1167/tvst.11.9.27>.

7. Haymes SA, Johnston AW, Heyes AD. The development of the Melbourne low-vision ADL index: A measure of vision disability. Invest Ophthalmol Vis Sci. 2001;42(6):1215-25.

8. Owsley C, Sloane M, McGwin G, Jr., Ball K. Timed instrumental activities of daily living tasks: Relationship to cognitive function and everyday performance assessments in older adults. Gerontology. 2002;48(4):254-65. <https://doi.org/10.1159/000058360>.

9. Dougherty BE, Martin SR, Kelly CB, Jones LA, Raasch TW, Bullimore MA. Development of a battery of functional tests for low vision. Optom Vis Sci. 2009;86(8):955-63. <https://doi.org/10.1097/OPX.0b013e3181b180a6>.

10. Latham K, Usherwood C. Assessing visual activities of daily living in the visually impaired. Ophthalmic Physiol Opt. 2010;30(1):55-65. <https://doi.org/10.1111/j.1475-1313.2009.00693.x>.

11. Ross CK, Stelmack JA, Stelmack TR, Guihan M, Fraim M. Development and sensitivity to visual impairment of the low vision functional status evaluation (LVFSE). Optom Vis Sci. 1999;76(4):212-20. <https://doi.org/10.1097/00006324-199904000-00024>.

12. Owsley C, McGwin G, Jr., Scilley K, Kallies K. Development of a questionnaire to assess vision problems under low luminance in age-related maculopathy. Invest Ophthalmol Vis Sci. 2006;47(2):528-35. <https://doi.org/10.1167/iovs.05-1222>.

13. Owsley C, McGwin G, Jr., Sloane ME, Stalvey BT, Wells J. Timed instrumental activities of daily living tasks: Relationship to visual function in older adults. Optom Vis Sci. 2001;78(5):350-9. <https://doi.org/10.1097/00006324-200105000-00019>.

14. Trauzettel-Klosinski S, Dietz K. Standardized assessment of reading performance: The new international reading speed texts IReST. Invest Ophthalmol Vis Sci. 2012;53(9):5452-61. <https://doi.org/10.1167/iovs.11-8284>.

15. Ying GS, Maguire MG, Liu C, Antoszyk AN. Night vision symptoms and progression of age-related macular degeneration in the complications of age-related macular degeneration prevention trial. Ophthalmology. 2008;115(11):1876-82. <https://doi.org/10.1016/j.ophtha.2008.05.023>.

16. Turano KA, Geruschat DR, Stahl JW, Massof RW. Perceived visual ability for independent mobility in persons with retinitis pigmentosa. Invest Ophthalmol Vis Sci. 1999;40(5):865-77.

17. Bierings R, van Sonderen FLP, Jansonius NM. Visual complaints of patients with glaucoma and controls under optimal and extreme luminance conditions. Acta Ophthalmol. 2018;96(3):288-94. <https://doi.org/10.1111/aos.13695>.

18. Wu Z, Guymer RH, Finger RP. Low luminance deficit and night vision symptoms in intermediate age-related macular degeneration. Br J Ophthalmol. 2016;100(3):395-8. <https://doi.org/10.1136/bjophthalmol-2015-306621>.

19. Alexander MS, Lajoie K, Neima DR, Strath RA, Robinovitch SN, Marigold DS. Effect of ambient light and age-related macular degeneration on precision walking. Optom Vis Sci. 2014;91(8):990-9. <https://doi.org/10.1097/OPX.0000000000000316>.

20. Bicket AK, Mihailovic A, E JY, Nguyen A, Mukherjee MR, Friedman DS, et al. Gait in elderly glaucoma: Impact of lighting conditions, changes in lighting, and fear of falling. Transl Vis Sci Technol. 2020;9(13):23. <https://doi.org/10.1167/tvst.9.13.23>.
